# A Resident’s Perspective of Ovarian Cancer

**DOI:** 10.3390/diagnostics7020024

**Published:** 2017-04-27

**Authors:** Christopher G. Smith

**Affiliations:** Department of Obstetrics & Gynecology, University of Kentucky Medical Center, 800 Rose Street, Lexington, KY 40536-0293, USA; christophergsmith@uky.edu

**Keywords:** ovarian cancer, screening, transvaginal ultrasound, quality of life

## Abstract

Identifying, understanding, and curing disease is a lifelong endeavor for any medical practitioner. Equally as important is to be cognizant of the impact a disease has on the individual suffering from it, as well as on their family. Ovarian cancer is the leading cause of death from gynecologic malignancies. Symptoms are vague, and the disease is generally at an advanced stage at diagnosis. Efforts have been made to develop methods to identify ovarian cancer at earlier stages, thus improving overall mortality. Transvaginal ultrasound (TVUS), with and without laboratory tests, can be used to screen for ovarian cancer. For over thirty years, the University of Kentucky Markey Cancer Center Ovarian Cancer Screening Program has been studying the efficacy of TVUS for detecting early stage ovarian cancer. After 285,000+ TVUS examinations provided to over 45,000 women, the program has demonstrated that regular TVUS examinations can detect ovarian cancer at early stages, and that survival is increased in those women whose ovarian cancer was detected with screening and who undergo standard treatment. These results demonstrate the utility of TVUS as an efficacious method of ovarian cancer screening.

## 1. Introduction

As a first-year resident in Obstetrics and Gynecology, I am relating my perspective on ovarian cancer at this stage of my career. This disease has received modest attention during medical school and during residency. Having been to weekly didactics dedicated to this disease, as well as opportunities to scrub in on complex pelvic surgeries, I am beginning to understand more about the nuances of this disease process, its diagnosis, and management. Much of what I am relating here I have learned in my own efforts to eventually prepare me for a career in Gynecologic Oncology.

Cancer is the second most common cause of death among women in the United States of America and is the leading cause of death among women 40 to 79 years of age. Among the types of cancers affecting women, ovarian cancer is considered uncommon, yet it causes severe morbidity and mortality [1]. Abdominal ascites, bowel obstructions, venous thromboses, and adverse effects from chemotherapy are the realities faced by women diagnosed with ovarian cancer. Ovarian cancer is most often diagnosed at an advanced stage, which has led to investigations of screening tests to detect the disease at early stages. Transvaginal ultrasonography (TVUS) has been studied as a means to characterize and categorize adnexal masses. Since 1987, the University of Kentucky Markey Cancer Center Ovarian Cancer Screening Program has been studying the efficacy of TVUS for detecting early stage ovarian cancer. After over 285,000+ TVUS examinations provided to over 45,000 women, the program has demonstrated that regular TVUS examinations can detect ovarian cancer at early stages, and that survival increased in those women whose ovarian cancer was detected with screening and underwent standard treatment [2,3].

Histologically, ovarian cancer is any neoplasm arising from ovarian cells. Historically, these cells can either be those that line the surface of the ovary (epithelial) or those that originate from the ovary as non-epithelial cancers (embryonic or extra-embryonic (germ), hormone-producing, or structural cells [sex-cord stromal]) [4,5,6,7,8]. In recent years, numerous reports have proposed a unified hypothesis about the origin of high-grade serous ovarian cancer, implicating the Fallopian tubes fimbria as the point of origin [9,10,11,12,13,14,15,16,17,18]. In this hypothesis, invasive or serous tubal intraepithelial carcinoma (STIC) originating in the Fallopian fimbria is responsible for seeding the ovaries and peritoneal cavity with malignant cells [19]. However, STIC is not present in many high-grade serous carcinomas [20].

Ovarian cancer generally affects older women, the average age being 63 [21]. Ovarian cancer is the eleventh most common cause of cancer among women, with a lifetime risk of one in 70 to develop disease [22]. It is also the leading cause of death from gynecological malignancy. Nearly two thirds of ovarian carcinomas are diagnosed with disease located outside of the pelvis and thereby impose the consequences of advanced stage disease. Overall, the five-year survival rate for women diagnosed with ovarian cancer is 46%. When ovarian cancer spreads to distant sites, five-year survival decreases to 28%, and decreases to nearly 16% with Stage 4 disease [1].

## 2. Ovarian Cancer Risk Factors

### 2.1. Inherent Risk Factors

Over her lifetime, a woman has a nearly one in 70 chance of developing ovarian cancer [17]. However, certain risk factors confer an increased chance of developing ovarian cancer. Nulligravidity, or never becoming pregnant, can increase the risk for ovarian cancer. The basis for the increased risk is that repetitive ovulation can cause cellular damage, inflammation, and cellular repair, all processes that increase the likelihood of introducing DNA mutations. Multiple pregnancies, using contraception methods that interrupt ovulation, and ovulation suppression due to extended lactation can reduce the risk of developing ovarian cancer [23].

### 2.2. Genetic Risk Factors

The most significant risk factor for ovarian cancer is a strong family history of gynecological, breast, or colon cancers. These women generally have an underlying genetic predisposition to developing ovarian cancer and have mutations in tumor suppressor genes that prevent cancer [24]. Mutations that lead to the loss of function of tumor suppressor genes are recessive; therefore, they must be passed on by both parents to their daughter, resulting in an increased risk of cancer. However, in the case of ovarian cancer, there are mutations that are dominant, so that only one copy of the mutated gene needs to be inherited from either parent. BRCA-1 and -2 are tumor suppressor genes, specifically caretaker genes, that encode proteins involved in DNA repair that prevent the accumulation of mistakes encoded in DNA [25,26]. Ovarian cancer associated with BRCA-1/2 mutations is more indolent and affects younger women. Among women with mutations in BRCA-1, the risk of ovarian cancer can range from 39% to 44%, while the risk with BRCA-2 mutations 12% to 20% [27,28,29].

Lynch Syndrome is a disorder that predisposes women to right sided non-polyposis colon cancer and ovarian and endometrial cancers. There are five tumor suppressor genes mutations associated with Lynch Syndrome: MSH2, MLH1, MLH6, PMS1, and PMS2. Mutations in these genes are inherited in a dominant fashion, and result in increased microsatellite instability, or regions of DNA with incorrectly transcribed DNA. Ovarian cancer risk in women with Lynch Syndrome is six to 8% [30]. It is quite clear that women with inherited genetic mutations are at a greater risk of developing ovarian cancer, with a nearly three to fifteen-fold increase in risk for different gene mutations [31].

## 3. Clinical Presentation

### 3.1. Symptoms

Ovarian cancer is considered a “silent killer”, meaning most women have no symptoms from the disease. Symptoms reported to be associated with ovarian cancer [32,33] are more often non-specific and associated with other conditions [26]. Sometimes, patients may present to their clinician with pelvic pain secondary to ovarian torsion. It is rare that any symptoms are associated with early stage ovarian cancer [34,35], and even when they do occur it is possible that they are coincidental. Women with advanced disease, however, are likely to have complaints of pelvic pain, abdominal fullness, early satiety, and bloating when tumor burden inflames abdominal structures.

### 3.2. Physical Examination Findings

A clinician may have an increased index of suspicion for ovarian cancer following their physical examination of the patient. A palpable pelvic mass, ascites with a fluid wave, or diminished breath sounds from pleural effusions can be identified on a physical examination. Rarely, a Sister-Mary Joseph nodule, resulting from ovarian cancer metastasized to the umbilicus, or the Sign of Leser-Trelat, which is an abrupt increase in seborrheic keratoses, can be indicative of occult cancer.

### 3.3. Ovarian Cancer Paraneoplastic Syndromes

Various paraneoplastic syndromes are infrequently associated with ovarian cancer. Hypercalcemia, usually due to increased levels of circulating parathyroid hormone releasing protein, can occur and cause altered mental status, increased thirst, urination, fatigue, constipation, and abdominal pain. Subacute cerebellar degeneration presenting as ataxia, dysarthria, vertigo, nystagmus, and double vision is due to cross-reactivity of antibodies to tumor antigens to cerebellar tissue. This condition usually precedes tumor occurrence by months to years, and can be associated with severe morbidity and mortality. Finally, Trousseau’s syndrome, or unexplained thromboses, has been associated with ovarian cancer [36].

## 4. Diagnosis of Ovarian Cancer

### 4.1. Diagnostic Schema

If there is a high clinical index of suspicion, diagnostic evaluation can be undertaken. This begins with a transvaginal ultrasound (TVUS). TVUS is highly sensitive and provides morphological information about the ovary. Abnormal cystic findings on TVUS are broadly defined as simple or complex, with echogenic components in complex cysts more indicative of malignancy. Ultrasound findings can then be paired with blood tests that measure levels of tumor markers.

### 4.2. Tumor Markers

Discovered over 30 years ago, CA-125 is one of the most utilized biomarkers for ovarian cancer [37,38]. When circulating levels of the CA-125 glycoprotein are elevated, it is often indicative of ovarian cancer, although benign conditions like pregnancy, menstruation, endometriosis, and pelvic inflammation can also be responsible for elevated CA-125 levels [39]. CA-125 can be used to calculate the risk of malignancy index (RMI) for an individual patient. The RMI consists of a score assigned to TVUS findings, menopausal status, and CA-125 level. RMI values greater than 200 indicate high risk of malignancy [40].

A biomarker reported to be more sensitive for identifying ovarian cancer is HE-4, which is expressed on multiple organs but, surprisingly, not on the ovary. Elevations in HE-4 are found in nearly 100% of serous and endometrioid ovarian cancers and are sensitive in diagnosing early ovarian cancer. Compared to CA-125, HE-4 is not elevated in benign processes, allowing the biomarker to be specific for ovarian malignancy. The caveat for utilizing HE-4 is that normal values are not established. With the high specificity of HE-4, and the high sensitivity of CA-125, the utility of combining the two for diagnosing ovarian cancer has been implemented as the Risk of Malignancy algorithm (ROMA). The ROMA uses a mathematical formula utilizing HE-4 and CA-125 concentrations adjusted for pre- and post-menopausal status. Elevated ROMA values place women in a high risk of malignancy category. The ROMA serves as a good screening test that also has specificity for epithelial ovarian cancer. It not only detects more patients with ovarian cancer than the RMI, but also those with early stages of ovarian cancer [41].

In 2009, the Food and Drug Administration approved the clinical use of OVA-1, a serum test analyzing five biomarkers: CA-125, II-microglobulin (both elevated in ovarian cancer), apolipoprotein A1, prealbumin (transthyretin), and transferrin (which are decreased in ovarian cancer). Biomarker levels are used in a computer algorithm to provide a result between zero and ten and are stratified based on menopausal status. Patients with higher scores should be evaluated by a gynecologic oncologist because the complexity of their disease is expected to be greater than those with lower scores. To date, there are no studies directly comparing the performance of OVA-1 and ROMA [42].

## 5. Ovarian Cancer Staging

Once a woman is considered to be at high risk for ovarian malignancy, a referral to a gynecologic oncologist is made. Surgery is generally undertaken to properly assess the extent of the disease. An exploratory laparotomy through a midline incision allows for gross evaluation of the abdominal and pelvic cavities for disease. If staging of ovarian cancer is found to be necessary, saline is initially used to irrigate the pelvis and collected as “pelvic washings”. This is followed by the surgical removal of the uterus, cervix, both Fallopian tubes, ovaries, lymph nodes that drain the ovaries (para-aortic lymph nodes), and the fat pad that insulates the intestines (omentum). Tissue is sent to the pathologist for final diagnosis of histological type, grade, and staging [31].

## 6. Treatment of Ovarian Cancer and Side Effects

### 6.1. Side Effects of Surgery

There are multiple side effects in the treatment of ovarian cancer. With surgery, potential risks generally include infection, hemorrhage, blood transfusion, pain, prolonged hospitalization, readmission, anesthesia complications, and death. Ovarian cancer surgical staging is often considered to be an “intermediate-complex surgery” and is associated with a 20% risk of morbidity and mortality occurring within the first 30 days following the operation [43]. Additionally, there is a ten to 15% risk of surgical site infections [44].

### 6.2. Chemotherapy and Associated Side Effects

Following surgery, most women receive some sort of chemotherapy treatment to eradicate any residual microscopic disease. More advanced stage ovarian cancer will have a greater likelihood of being associated with residual disease. Various platinum-based chemotherapy regimens have been used and are dependent on the stage of the cancer. These agents are not without side effects that can include nausea, renal and ototoxicity, myalgia, alopecia, bone marrow toxicity with resulting pancytopenia, mouth sores, swelling, redness, and chronic pain in the hands and feet (hand-foot syndrome) [45,46].

## 7. Psychosocial Effects of Ovarian Cancer

There can be considerable emotional and physical burdens associated with ovarian cancer. Anxiety and depression can develop from the distress over the pending removal of organs that represent a woman’s femininity, motherhood, and sexuality [47]. Recurrence of disease is common, nearly 80% with advanced stage, serving as another nidus for stress. In a study from the Dana-Farber Cancer Institute, 56% of ovarian cancer survivors surveyed were concerned about recurrence [48]. Anxiety and depression is nearly two times more likely in women with ovarian cancer, and higher if there are other underlying health issues. Additionally, nearly 33% of ovarian cancer patients experience high levels of psychological distress [49]. The burden of ovarian cancer can be extended to caregivers. The Australian Ovarian Cancer Study Group investigated the effects of ovarian cancer on the quality of life of ovarian cancer caregivers. This study found that in the last year of life, caregivers had lower quality of life measures as well as higher distress than those who were not taking care of ovarian cancer partners. Additionally, mental and physical well-being worsened the closer their partner came to the end-of-life. The most reported unmet needs of caregivers in the last six months were found to be concerned with managing emotions surrounding prognosis, fear of worsening disease, balancing of both the needs of themselves and their partners, the impact of caring for their partner had on their career, and making decisions in an environment of uncertainty [50].

## 8. Ovarian Cancer Screening

### 8.1. Overview of Early Detection of Ovarian Cancer

Advanced ovarian cancer is associated with decreased survival, and increased morbidity with not only the disease itself, but also surgery and the effects of chemotherapy. A recent study found that if 75% of ovarian cases can be detected as Stage I or II disease, there would be a 50% reduction in ovarian cancer related deaths [3]. Therefore, there have been multiple investigations to improve detection of ovarian at earlier stages.

### 8.2. Disease Screening Principles

One method is to screen women for ovarian cancer. Disease that benefits from screening is one that is (1) highly prevalent in the population, (2) a major health problem, (3) has a significant preclinical stage during which detection by screening is possible, and (4) is significantly more curable at earlier stages. Ovarian cancer satisfies these conditions, but does challenge the condition of prevalence.

Screening for a disease is the process by which an asymptomatic population is evaluated for the likelihood of having the disease before there are symptoms or any indication of disease. Screening has two outcomes: positive (likely has the disease) or negative (does not have the disease). The ideal screening test will have:

- High sensitivity: the ability to identify everyone with disease who tests positive (true positive) from everyone with disease (true positives + false negatives). Ideally, a highly sensitive test will have a low rate of false negative results so the test rarely misses subjects with the disease;
- High specificity: the ability to correctly identify subjects *without* the disease (true negatives) from everyone without disease (true negatives + false positives). A highly specific screening test will have a low false positive rate;
- High positive predictive value: the portion of subjects with disease that tested positive (true positives) relative to all who tested positive (true positives + false positives), a value dependent on the prevalence of the disease;
- High negative predictive value: the portion without disease that tested negative (true negatives) relative to everyone testing negative (true negatives + false negatives), which is *inversely* dependent on disease prevalence;
- Low cost: to allow maximum test affordability.

Table 1 summarizes these terms and relates them to ovarian cancer screening.

Screening tests are compared to an acceptable “gold standard” test, which is usually a definitive diagnostic test. It is typically invasive, unpleasant, expensive, or impractical for wide use. Considered the best test under “reasonable conditions”, the “gold standard” test provides 100% sensitivity and specificity [52]. Regarding ovarian cancer, there is no current “gold standard” screening test. However, TVUS performs with the highest sensitivity and specificity. A TVUS is performed with a 5–7.5 mHz vaginal probe that generates accurate images of the ovary used to detect changes in ovarian morphology and volume that are subtle and usually inappreciable on physical examination.

### 8.3. Ovarian Cancer Screening Trials

#### 8.3.1. University of Kentucky Ovarian Cancer Screening Program

**Figure 1:**
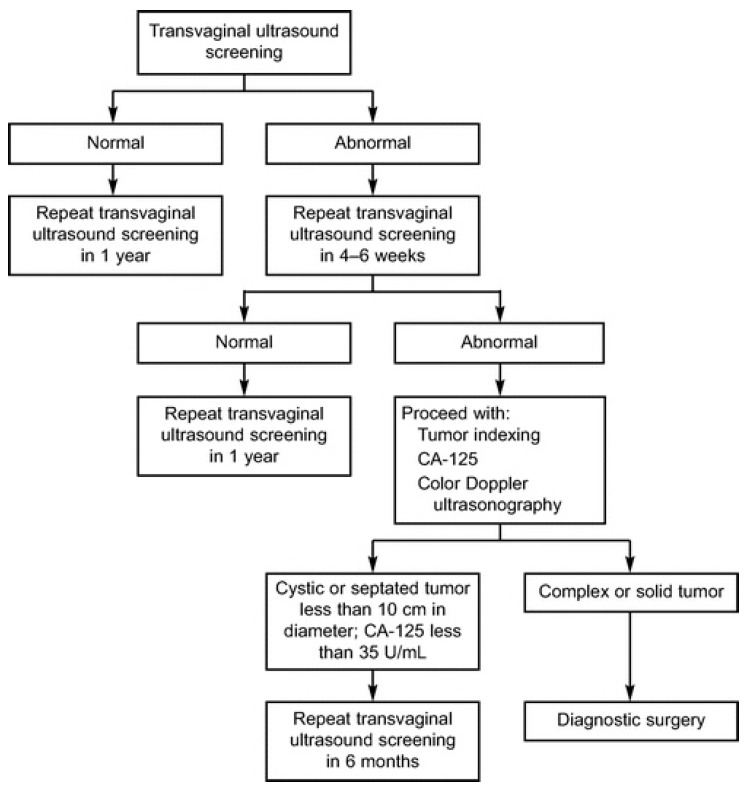
University of Kentucky Ovarian Cancer Screening Trial screening, evaluation, and treatment algorithm [53]. Reproduced with permission from publisher.

There have been four large ovarian cancer screening trials with TVUS as the primary screening modality, one of which is the University of Kentucky Markey Cancer Center Ovarian Cancer Screening Program. Originally initiated in 1987 by Dr. John R. van Nagall, this project has enrolled over 45,000 women, and investigators have performed over 280,000 scans. The project includes two groups: asymptomatic women ≥50 years old, and asymptomatic women ≥25–49 years old with a documented history of ovarian cancer in at least one primary or secondary family member. Both groups of women are compared to an unscreened control group of women from the same geographic area who received the same treatment protocols over the same period. The groups undergoing screening undergo evaluation based on an established algorithm (Figure 1).

Using the algorithm, the detection of 53 primary epithelial ovarian malignancies has been reported [2]. Women who had ovarian cancer diagnosed by screening had earlier-stage disease (Stage 1 or 2) than those who did not receive screening (68% vs. 27%). The five-year survival rate of all women whose ovarian cancer was detected by screening compared to those not undergoing screening was 74.8% ± 6.6% and 53.7% ± 2.3%, a statistically significant difference. To date, the overall sensitivity, specificity positive and negative predictive values, and false positive rate are 86.4%, 98.8%, 14.53% to 20.17%, 99.97%, and 1.2%, respectively [54,55,56,57].

#### 8.3.2. Prostate, Lung, Colon, and Ovarian Cancer Screening Trial

The Prostate, Lung, Colon, and Ovarian Cancer Screening Trial (PLCO) is a large, population-based randomized trial designed and sponsored by the National Cancer Institute starting in 1993 to determine the effects of screening on cancer-related mortality and secondary outcomes in men and women aged 55 to 74. Regarding ovarian cancer screening, women were assigned to undergo either annual screening with CA-125 and TVUS or usual care. There was no statistically significant reduction in ovarian cancer–related deaths between those screened and those who underwent usual care. There was a minimal increase in the detection of early stage ovarian cancer with screening than with usual care (22% vs. 21%). The five-year survival rate was 47.4% in the screening group compared to 36.0% in the group receiving usual care. The sensitivity, specificity, positive and negative predictive values, and false positive rate were 85.14%, 90.34%, 6.06%, 99.88%, and 9.6%, respectively [58,59].

#### 8.3.3. United Kingdom Collaborative Trial of Ovarian Cancer Screening Trial

The United Kingdom Collaborative Trial of Ovarian Cancer Screening (UKCTOCS) trial recruited 200,000 women beginning in 2001 and randomized them into a usual care group, annual screening with TVUS group, or annual multimodal screening with CA-125 and risk for ovarian cancer algorithm (ROCA) group. The ROC utilizes an individual’s CA-125 level profile (initial values and trends over time) and compares it to populations of women with and without cancer. The more a woman’s ROC looks like profiles of women who have ovarian cancer, the greater her risk of having ovarian cancer [60]. Women found to have a high ROC scores were subsequently screened with TVUS per their algorithm. Results demonstrated an increased detection of low-volume disease (Stage 1, 2, and 3a) in the multimodal screening group than by TVUS alone (40% vs. 24%). There was no reduction in mortality regardless of the type of screening. Regarding the detection of any primary ovarian, tubal, or peritoneal cancers, the sensitivity, specificity, and positive predictive value for the TVUS group were found to be 84.9%, 98.2%, and 5.3%, respectively. The multimodal screening had a sensitivity of 89.4%, specificity of 99.8%, and a positive predictive value of 43.3%, respectively. When detecting primary invasive ovarian, tubal, and peritoneal malignancies, the sensitivity and positive predictive value of TVUS decreased to 75% and 2.8%. The multimodal screening was essentially unchanged, yet the positive predictive value decreased to 35% [61,62].

#### 8.3.4. Multi-Center Japan University Trial

The final, large-scale ovarian cancer screening trial is the Multi-center Japan University trial. Over 80,000 asymptomatic women were divided into either a control group consisting of usual care following a physical examination or a screening group. Screening involved an annual pelvic examination, TVUS and/or transabdominal ultrasound, and measurement of CA-125 levels. The study showed that screening could detect Stage I and II disease at a higher rate compared to usual care (67% and 44%, respectively). Using the published data, the sensitivity, specificity, and positive and negative predictive values were 68.6%, 99.8%, 23.3% and 99.9%, respectively. The analysis of this screening protocol for the long-term effect on ovarian cancer mortality is presently in progress [63].

## 9. Life Is More Than Death: An Interview with the Husband of a Recently Deceased Woman Suffering from Advanced Stage Ovarian Cancer

In my short time in medical school and residency, I have made myself familiar with the pathophysiology of ovarian cancer, its treatments, the status of screening for the disease, and the survival curves that serve as the ultimate sterile summary of this disease. In all actuality, survival curves are the measure of time from diagnosis to death, conveying nothing more than the math of mortality. I have looked into the faces of women at various stages of this disease and have seen the suffering in their eyes as their mortality approaches them.

I felt compelled to convey only one story, a story that is much larger than the deaths due to ovarian cancer, because it is the journey on the terrifying road that women travel from their diagnosis to their death. I received permission to discuss the final stages of ovarian cancer with the husband and subsequent caregiver of a patient who died from ovarian cancer. To preserve patient confidentiality, identities are de-identified with pseudonyms.

### 9.1. Pre-Diagnosis Life

Mr. and Mrs. Johnson (pseudonyms) were college sweethearts who met through a mutual friend. Their first date consisted of a lovely bike ride across their college campus. Eventually, they wed and had three children. Mrs. Johnson primarily took care of their children and homeschooled them much of the time. Eventually, the children grew up and left home. She enjoyed working with her hands and loved embroidery, designing children’s clothing, and creating smocking designs.

Mrs. Johnson took very good care of herself and never missed her annual gynecologic examinations. However, one day she noticed changes in her body. She mentioned this to her husband and said she “felt like she was filling up with water”. Initially, they dismissed the complaint as something that would resolve itself. However, two days later, she again commented on how bloated she felt, and lifted her shirt, showing her husband how distended her abdomen was. She began to shake her hips, and they both could hear “water sloshing around her abdomen”. Mrs. Johnson called a physician friend, who urged her to set up an appointment with her gynecologist.

At the discretion of her gynecologist, she underwent a CT scan and had a CA-125 level drawn. When her doctor entered the room with the results, he “had a complete change in his demeanor”. Her CA-125 was over 15,000, and imaging showed some sort of gynecologic malignancy. Her gynecologist counseled her that she needed to be evaluated by a gynecologic oncologist and suggested she be evaluated at the Markey Cancer Center, located on the University of Kentucky Medical Center campus.

The gynecologic oncologist was certain she was suffering from at least Stage IIIC ovarian cancer and that a staging surgery was necessary. Surgery revealed extensive disease throughout the abdomen including involvement of the liver and diaphragm. A ureter obstruction required a ureter re-anastomosis by a urology consultation team. Post-operatively, she spent 11 days in the hospital, which was complicated by a right pleural effusion. The final pathology was consistent with high-grade Stage IVA papillary serous adenocarcinoma of the ovary arising from both ovaries. According to her husband, revelation of her diagnosis was “like being hit between the eyes”.

### 9.2. Life after Diagnosis for Both Individuals

Though the chances of a five-year survival had been quoted as 15%, Mrs. Johnson had always been a strongminded individual, and she was determined to beat her disease. Chemotherapy options were discussed, and she elected to proceed with dose-dense carboplatin and taxol.

### 9.3. Life during Treatment for Both Individuals

Life during chemotherapy was a struggle, though she did not let those struggles dampen her faith and determination. Fatigue was the worst side effect from her chemotherapy to the point that she was unable to enjoy her usual embroidery activities. Faith played a large role in keeping her will to beat cancer alive. Her family and church members prayed constantly that she would not suffer from neuropathy in her hands so she could continue knitting, and thankfully, their prayers were answered. She had mild neuropathy in her feet with sparing of her hands.

Mrs. Johnson experienced recurrent pleural effusions, eventually showing evidence of malignancy and recurrence. She had the the lining of her right hemi-thorax removed, which did not show evidence of disease. Additionally, a new area of suspected malignancy on her spleen was evident on a repeat CT scan, along with a rise in her CA-125 to a maximum level of 4000. She received various chemotherapy regimens including, gemcitabine, Avastin with vinorelbine, taxotere, cyclophosphamide, and etoposide. External beam radiation therapy was utilized to address the malignant area of her spleen. She had 13 radiation treatments, but decided to decline any more radiation treatments because they caused more fatigue than her chemotherapy. Her cancer responded to the cyclophosphamide, but eventually her CA-125 began to rise. She declined further chemotherapy, and the topic of hospice was approached. She decided that she did not want hospice at that point.

### 9.4. Life during the Final Months for Both Individuals

The final months of Mrs. Johnson’s life were filled with overwhelming difficulties resulting from her recurrent ovarian cancer. She suffered greatly from recurrent pleural effusions and abdominal ascites. She had nearly eight liters of ascites removed as an outpatient. She would continue to have paracenteses whenever she became symptomatic. She decided to resume chemotherapy treatment with etoposide. Two days later, she was admitted to the hospital with chemotherapy induced nausea and vomiting. She was discharged after five days, but was readmitted for intractable symptoms. She wanted to have a Denver drain placed to allow personal drainage of her ascites whenever she was symptomatic. This improved her quality of life considerably.

However, she suffered another setback when she was diagnosed with a small bowel obstruction. She elected to forego aggressive surgical treatment to address the obstruction and decided instead to go home to be treated with intravenous fluids. The topic of hospice was brought up multiple times, but she did not want to give in to her cancer. She was unable to tolerate any food, but she was determined to eat. The thought of food became an obsession, as she would “watch cooking shows and read every page of every cookbook in her kitchen”. She developed a routine of self-induced emesis in the morning and evenings so that she could at least eat something. She continued to be symptomatic from her small bowel obstruction, utilizing outpatient intravenous fluid hydration and electrolyte replacements. Through all of this, she still declined hospice, with hope still alive of overcoming her disease.

Mrs. Johnson died in November 2016. From the time of diagnosis, she lived 33 months, underwent 66 chemotherapy treatments with 10 different chemotherapy agents, 13 external radiation treatments, five thoracenteses to relieve recurrent pleural effusions, and four paracentesis procedures that removed nearly eight liters of ascites. Mrs. Johnson did not live without pain, nor die without immense suffering.

### 9.5. Feelings of the Family (Husband) after the Woman’s Death 

Mrs. Johnson’s family had a very peaceful Thanksgiving with her son and his wife assuming the cooking responsibilities that Mrs. Johnson had traditionally performed. Christmas was “a ‘new’ Christmas, but not a sad one”. Mr. Johnson said that he did not regret anything about his wife’s fight with cancer, and though it was the most terrible time for them, they would do nothing different if they had to go through it again.

## 10. Conclusions

It is estimated that a woman’s lifetime risk of developing ovarian cancer is one in 70. Many ovarian cancers are diagnosed at an advanced stage, with only 15% of cases diagnosed in early stages. Ovarian cancer screening trials have attempted to diagnose women with early stage disease because survival is significantly greater in those patients. With screening programs like the University of Kentucky Markey Cancer Center Ovarian Cancer Screening Program, transvaginal ultrasound is effective for discovering early stage ovarian cancer. With continued efforts and determination, more ovarian cancers can be diagnosed at earlier, more curable stages, avoiding the pain and suffering associated with advanced stage disease like that endured by Mrs. Johnson.

## Figures and Tables

**Table 1:** Statistical terms and definitions used in ovarian cancer screening [51].

Term	Screening Result	Findings
True Positive (TP)	Positive	Histologically-proven ovarian cancer
False Positive (FP)	Positive	Benign ovarian histology
True Negative (TN)	Negative	No evidence of ovarian cancer 12 months after a negative screen
False Negative (FN)	Negative	Ovarian cancer diagnosed within 12 months of a negative screen

Sensitivity = TP/(TP + FN); Specificity = TN/(TN + FP); Positive Predictive Value = TP/(TP + FP); Negative Predictive Value = TN/(TN + FN). Reproduced from [51] with permission from publisher.
